# Crystal structure and Hirshfeld surface analysis of (*E*)-1-[2,2-di­bromo-1-(2-nitro­phen­yl)ethen­yl]-2-(4-fluoro­phen­yl)diazene

**DOI:** 10.1107/S205698902200278X

**Published:** 2022-03-17

**Authors:** Sevim Türktekin Çelikesir, Mehmet Akkurt, Namiq Q. Shikhaliyev, Naila A. Mammadova, Gulnar T. Suleymanova, Victor N. Khrustalev, Ajaya Bhattarai

**Affiliations:** a Department of Physics, Faculty of Sciences, Erciyes University, 38039 Kayseri, Turkey; b Organic Chemistry Department, Baku State University, Z. Khalilov str. 23, AZ 1148 Baku, Azerbaijan; c Peoples’ Friendship University of Russia, 6 Miklukho-Maklaya, Moscow, Russian Federation; d N.D. Zelinsky Institute of Organic Chemistry, Russian Academy of Sciences, 47 Leninsky Av., Moscow, Russian Federation; e Department of Chemistry, M.M.A.M.C (Tribhuvan University) Biratnagar, Nepal

**Keywords:** crystal structure, C—F⋯π inter­action, π–π stacking inter­action, Hirshfeld surface analysis

## Abstract

In the title compound, C_14_H_8_Br_2_FN_3_O_2_, the nitro-substituted benzene ring and the 4-fluoro­phenyl ring form a dihedral angle of 65.73 (7)°. In the crystal, mol­ecules are linked into chains by C—H⋯O hydrogen bonds running parallel to the *c*-axis direction. The crystal packing is consolidated by C—F⋯π inter­actions and π–π stacking inter­actions, and short Br⋯O [2.9828 (13) Å] contacts are observed. The Hirshfeld surface analysis of the crystal structure indicates that the most important contributions to the crystal packing are from H⋯H (17.4%), O⋯H/H⋯O (16.3%), Br⋯H/H⋯Br (15.5%), Br⋯C/C⋯Br (10.1%) and F⋯H/H⋯F (8.1%) contacts.

## Abstract 2

The packing of the title compound features C—H⋯O hydrogen bonds, C—F⋯π inter­actions, aromatic π–π stacking and short Br⋯O contacts.

## Chemical context

Azo dyes are chemical compounds with the general formula *R*—N=N—*R*′, where *R* and *R*′ can be either aryl, hetrocycle or alkyl functional groups. They find many applications such as mol­ecular switches, optical data storage, anti­microbial agent, colour-changing materials, non-linear optics, mol­ecular recognition, dye-sensitized solar cells, liquid crystals, catalysis, *etc*. (see, for example, Kopylovich *et al.*, 2012 ▸; MacLeod *et al.*, 2012 ▸; Viswanathan *et al.*, 2019 ▸). Both *E*/*Z* isomerization and azo*-*to-hydrazo tautomerization of azo dyes is an important feature in the synthesis and design of new functional materials (Mahmudov *et al.*, 2012 ▸, 2020 ▸; Mizar *et al.*, 2012 ▸). On the other hand, the attachment of non-covalent bond-donor or acceptor centres to the azo dyes can be used as a synthetic strategy for the improvement of the functional properties of this class of organic compounds (Gurbanov *et al.*, 2020*a* ▸,*b* ▸). 
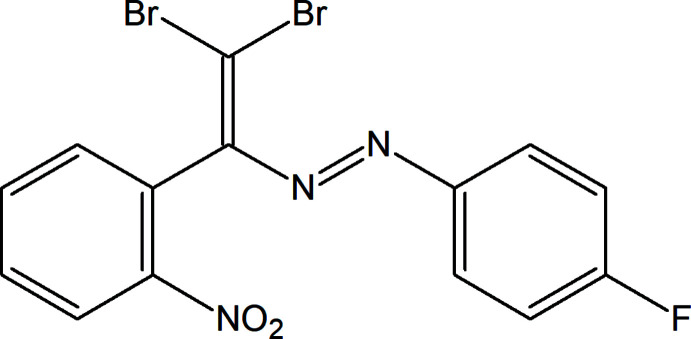


As part of our ongoing work in this area we have attached –F, –Br and –NO_2_ functional groups and aryl rings to the —N=N— moiety, leading to the title compound, C_14_H_8_Br_2_FN_3_O_2_, and determined its crystal structure.

## Structural commentary

**Figure 1:**
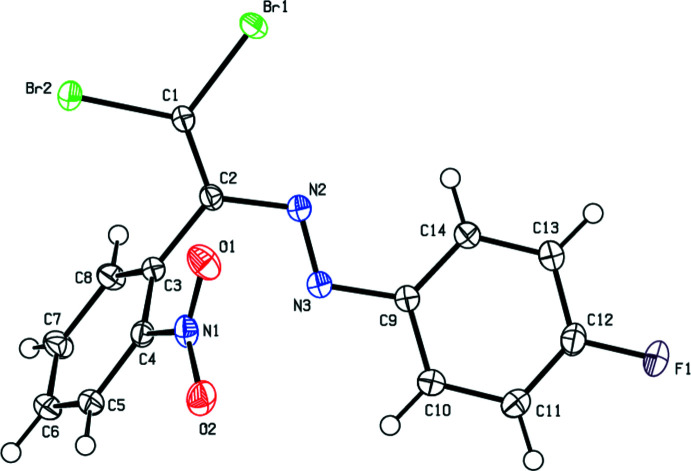
The mol­ecular structure of the title compound with displacement ellipsoids drawn at the 50% probability level.

As shown in Fig. 1 ▸, the mol­ecular conformation of the title compound is not planar, the nitro-substituted benzene ring and the 4-fluoro­phenyl ring forming a dihedral angle of 65.73 (7)°. There is a slight twist about the C1=C2 double bond with the dihedral angle between C1/Br1/Br2 and C2/C3/N2 being 3.35 (15)°, perhaps to minimize steric repulsion between Br2 and H8. Considered together, the N3/N2/C2/C1/Br1/Br2 moiety subtends dihedral angles of 70.40 (7) and 14.14 (7)° with the C3–C8 and C9–C14 rings, respectively. In the mol­ecule, the aromatic ring and olefin synthon adopt a *trans*-configuration with respect to the N=N double bond and are almost coplanar with a C2—N2=N3—C9 torsion angle of −178.50 (11)°. All of the other bond lengths and angles in the title compound are similar to those in the related azo compounds reported in the *Database survey*.

## Supra­molecular features

**Figure 2:**
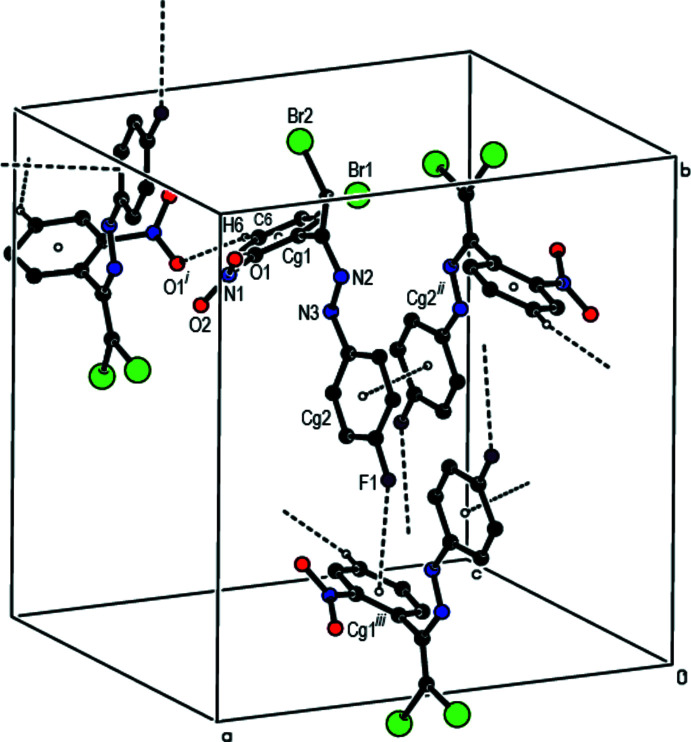
View of the C—H⋯O, C—F⋯π and π–π stacking inter­actions in the title compound.

In the crystal, mol­ecules are linked by C—H⋯O hydrogen bonds into chains propagating parallel to the c axis (Table 1 ▸; Fig. 2 ▸). The crystal packing is consolidated by weak C—F⋯π [F1⋯*Cg*1(*x*, 1 − *y*, − 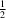 + *z*) = 3.4095 (12) Å; C—F⋯*Cg*1 = 136.95 (9)°] inter­actions and weak aromatic π–π stacking [*Cg*2⋯*Cg*2(1 − *x*, *y*, 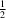 − *z*) = 3.9694 (9) Å], where *Cg*1 and *Cg*2 are the centroids of the C3–C8 and C9–C14 rings, respectively (Fig. 2 ▸). In addition, short bromine–oxygen contacts [Br2⋯O2( 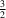 − *x*, 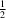 + *y*, *z*) = 2.9828 (13) Å; van der Waals contact distance = 3.37 Å] are observed.

## Hirshfeld surface analysis

**Figure 3:**
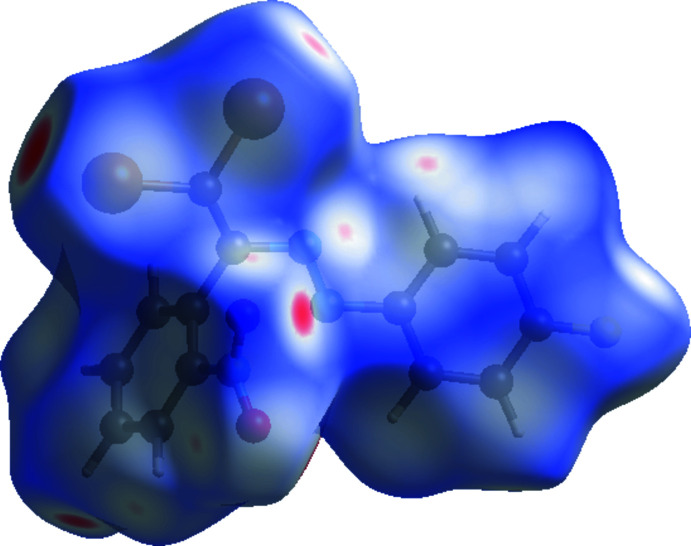
The three-dimensional Hirshfeld surface of the title compound plotted over *d*_norm_ in the range −0.24 to 1.44 a.u.

*CrystalExplorer17* (Turner *et al.*, 2017 ▸) was used to calculate the Hirshfeld surfaces for the title compound and generate the two-dimensional fingerprint plots. On the *d*_norm_ surface, red, white, and blue regions indicate contacts with distances shorter, longer, and roughly equal to the van der Waals radii for the title compound (Fig. 3 ▸, Tables 1 ▸ and 2 ▸).

**Figure 4:**
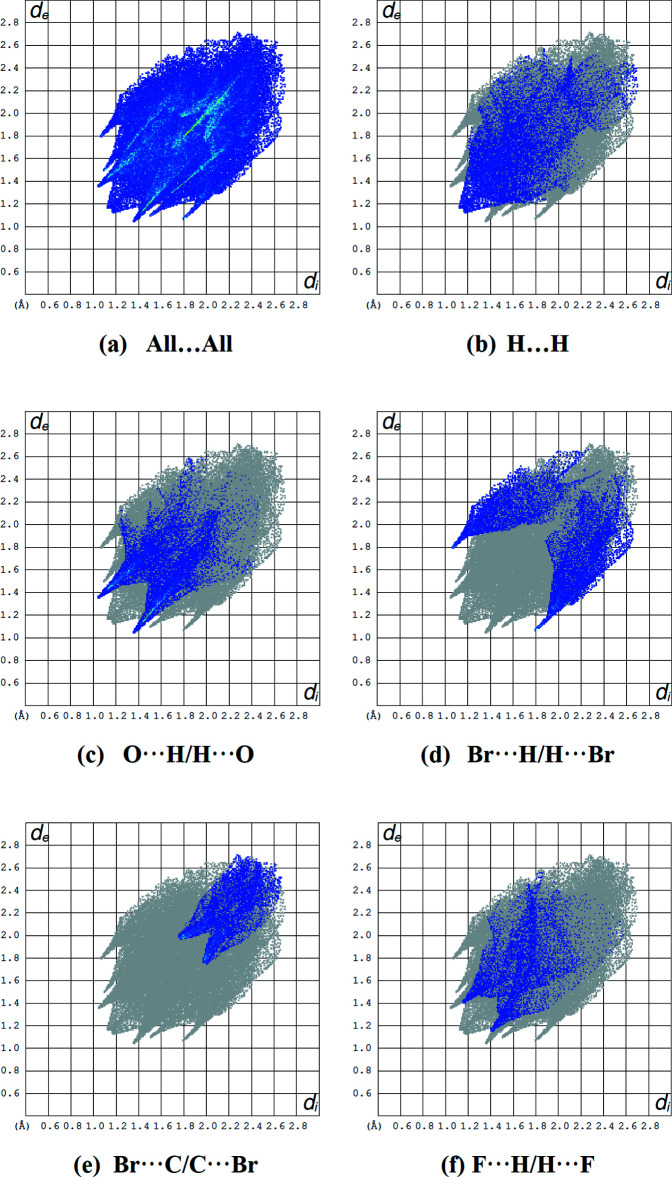
The full two-dimensional fingerprint plot (*a*) for the title compound and those delineated into (*b*) H⋯H (17.4%), (*c*) O⋯H/H⋯O (16.3%), (*d*) Br⋯H/H⋯Br (15.5%), (*e*) Br⋯C/C⋯Br (10.1%) and (*f*) F⋯H/H⋯F (8.1%) inter­actions.

The overall two-dimensional fingerprint plot (Fig. 4 ▸ *a*) and those delineated into H⋯H, O⋯H/H⋯O, Br⋯H/H⋯Br, Br⋯C/C⋯Br and F⋯H/H⋯F contacts (McKinnon *et al.*, 2007 ▸) are illustrated in Fig. 4 ▸ *b*–*f*, respectively. The most important inter­action is H⋯H, contributing 17.4% to the overall surface, which is reflected in Fig. 4 ▸ *b* as widely scattered points of high density due to the large hydrogen content of the mol­ecule, with the tip at *d*_e_ = *d*_i_ = 1.15 Å. The reciprocal O⋯H/H⋯O inter­actions appear as two symmetrical broad wings with *d*_e_ + *d*_i_ ≃ 2.40 Å and contribute 16.3% to the Hirshfeld surface (Fig. 4 ▸ *c*). In the Br⋯H/H⋯Br fingerprint plot, there are two symmetrical wings with *d*_e_ + *d*_i_ ≃ 2.85 Å and they contribute 15.5% to the Hirshfeld surface (Fig. 4 ▸ *d*). The pair of characteristic wings in the fingerprint plot delin­eated into Br⋯C/C⋯Br contacts (Fig. 8*e*; 10.1% contribution to the Hirshfeld surface), have the tips at *d*_e_ + *d*_i_ ≃ 3.80 Å, while for F⋯H/H⋯F contacts (Fig. 4 ▸ *f*; 8.1% contribution to the Hirshfeld surface), they have the tips at *d*_e_ + *d*_i_ ≃ 2.60 Å. The remaining contributions from the other different inter­atomic contacts to the Hirshfeld surfaces are listed in Table 3 ▸. The dominance of H-atom contacts suggest that van der Waals inter­actions play the major role in establishing the crystal packing for the title compound (Hathwar *et al.*, 2015 ▸).

## Database survey

A search of the Cambridge Structural Database (CSD, Version 5.42, update of September 2021; Groom *et al.*, 2016 ▸) for the (*E*)-1-(2,2-di­chloro-1-phenyl­ethen­yl)-2-phenyl­diazene unit gave 26 hits. Seven compounds are closely related to the title compound, *viz*. CSD refcode GUPHIL (**I**) (Özkaraca *et al.*, 2020 ▸), HONBUK (**II**) (Akkurt *et al.*, 2019 ▸), HONBOE (**III**) (Akkurt *et al.*, 2019 ▸), HODQAV (**IV**) (Shikhaliyev *et al.*, 2019 ▸), XIZREG (**V**) (Atioğlu *et al.*, 2019 ▸), LEQXOX (**VI**) (Shikhaliyev *et al.*, 2018 ▸) and LEQXIR (**VII**) (Shikhaliyev *et al.*, 2018 ▸).

In the crystal of (**I**), mol­ecules are linked into inversion dimers *via* short halogen–halogen contacts [Cl1⋯Cl1 = 3.3763 (9) Å C16—Cl1⋯Cl1 = 141.47 (7)°] compared to the van der Waals radius sum of 3.50 Å for two chlorine atoms. No other directional contacts could be identified and the shortest aromatic-ring-centroid separation is greater than 5.25 Å. In the crystals of (**II**) and (**III**), the aromatic rings form dihedral angles of 64.1 (2) and 60.9 (2)°, respectively. Mol­ecules are linked through weak *X*⋯Cl contacts [*X* = Cl for (**II**) and Br for (**III**)], C—H⋯Cl and C—Cl⋯π inter­actions into sheets lying parallel to the *ab* plane. In the crystal of (**IV**), the planes of the benzene rings make a dihedral angle of 56.13 (13)°. Mol­ecules are stacked in columns along the *a*-axis direction *via* weak C—H⋯Cl hydrogen bonds and face-to-face π–π stacking inter­actions. The crystal packing is further consolidated by short Cl⋯Cl contacts. In (**V**), the benzene rings form a dihedral angle of 63.29 (8)°. Mol­ecules are linked by C—H⋯O hydrogen bonds into zigzag chains running along the *c*-axis direction. The crystal packing also features C—Cl⋯π, C—F⋯π and N—O⋯π inter­actions. In the crystals of (**VI**) and (**VII**), the dihedral angles between the aromatic rings are 60.31 (14) and 56.18 (12) °, respectively. In (**VI**) C—H⋯N and short Cl⋯Cl contacts are observed and in (**VII**), C—H⋯N and C—H⋯O hydrogen bonds and short Cl⋯O contacts occur.

## Synthesis and crystallization

A 20 ml screw-neck vial was charged with DMSO (10 ml), (*E*)-1-(4-fluoro­phen­yl)-2-(2-nitro­benzyl­idene)hydrazine (1 mmol), tetra­methyl­ethylenedi­amine (TMEDA) (295 mg, 2.5 mmol), CuCl (2 mg, 0.02 mmol) and CBr_4_ (4.5 mmol). After 1–3h (until TLC analysis showed complete consumption of corresponding Schiff base) the reaction mixture was poured into a ∼0.01 *M* solution of HCl (100 ml, pH = 2–3), and extracted with di­chloro­methane (3 × 20 ml). The combined organic phase was washed with water (3 × 50 ml), brine (30 ml), dried over anhydrous Na_2_SO_4_ and concentrated *in vacuo* using a rotary evaporator. The residue was purified by column chromatography on silica gel using appropriate mixtures of hexane and di­chloro­methane (3/1–1/1). Crystals suitable for X-ray analysis were obtained by slow evaporation of an ethanol solution. Light-orange solid (52%); m.p. 377 K. Analysis calculated for C_14_H_8_Br_2_FN_3_O_2_ (*M* = 429.04): C 39.19, H 1.88, N 9.79; found: C 39.14, H 1.87, N 9.73%. ^1^H NMR (300MHz, CDCl_3_) *δ* 7.86–7.14 (8H, Ar–H). ^13^C NMR (75MHz, CDCl_3_) *δ* 165.02, 163.23, 163.01, 149.72, 133.01, 132.10, 129.70, 124.98, 124.87, 124.80, 124.29, 116.07, 115.91, 86.88. ESI–MS: *m*/*z*: 430.02 [*M* + H]^+^.

## Refinement details

Crystal data, data collection and structure refinement details are summarized in Table 4 ▸. All H atoms were positioned geometrically [C—H = 0.95 Å] and refined using a riding model with *U*_iso_(H) = 1.2*U*_eq_(C).

## Supplementary Material

Crystal structure: contains datablock(s) I. DOI: 10.1107/S205698902200278X/hb8012sup1.cif

Structure factors: contains datablock(s) I. DOI: 10.1107/S205698902200278X/hb8012Isup2.hkl

Supporting information file. DOI: 10.1107/S205698902200278X/hb8012Isup3.cml

CCDC reference: 2158375

Additional supporting information:  crystallographic information; 3D view; checkCIF report

## Figures and Tables

**Table 1:** Hydrogen-bond geometry (Å, °)

*D*—H⋯*A*	*D*—H	H⋯*A*	*D*⋯*A*	*D*—H⋯*A*
C6—H6⋯O1^i^	0.95	2.51	3.3244 (18)	144

Symmetry code: (i) 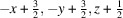 .

**Table 2:** Summary of short inter­atomic contacts (Å) in the title salt

Contact	Distance	Symmetry operation
H8⋯Br1	2.99	1 − *x*, *y*, 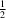 − *z*
O1⋯H11	2.68	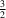 − *x*, 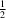 + *y*, *z*
Br1⋯Br2	3.6164	*x*, 2 − *y*, − 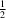 + *z*
H7⋯Br2	3.19	1 − *x*, 2 − *y*, 1 − *z*
H13⋯F1	2.82	1 − *x*, 1 − *y*, −*z*
F1⋯H10	2.67	*x*, 1 − *y*, − 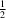 + *z*
O1⋯H6	2.51	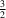 − *x*, 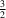 − *y*, − 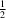 + *z*
O2⋯H8	2.77	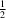 + *x*, 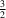 − *y*, 1 − *z*
H7⋯H6	2.47	1 − *x*, *y*, 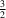 − *z*

**Table 3:** Percentage contributions of inter­atomic contacts to the Hirshfeld surface for the title salt

Contact	Percentage contribution
H⋯H	17.4
O⋯H/H⋯O	16.3
Br⋯H/H⋯Br	15.5
Br.·C/C⋯Br	10.1
F⋯H/H⋯F	8.1
C⋯H/H⋯C	7.0
N⋯H/H⋯N	5.5
C⋯C	4.7
Br.·O/O⋯Br	4.2
F⋯C/C⋯F	3.5
Br⋯Br	3.1
N⋯C/C⋯N	1.4
Br⋯F/F⋯Br	1.1
N⋯N	0.9
O⋯C/C⋯O	0.1
F⋯O/O⋯F	0.6
F⋯N/N⋯F	0.5

**Table 4:** Experimental details

Crystal data	
Chemical formula	C_14_H_8_Br_2_FN_3_O_2_
*M*_r_	429.05
Crystal system, space group	Orthorhombic, *P* *b* *c* *n*
Temperature (K)	100
*a*, *b*, *c* (Å)	14.8700 (4), 15.2915 (4), 13.1030 (4)
*V* (Å^3^)	2979.42 (14)
*Z*	8
Radiation type	Mo *K*α
μ (mm^−1^)	5.46
Crystal size (mm)	0.59 × 0.26 × 0.20

Data collection	
Diffractometer	Bruker AXS D8 QUEST Photon III detector
Absorption correction	Multi-scan (*SADABS*; Krause *et al.*, 2015 ▸)
*T*_min_, *T*_max_	0.047, 0.115
No. of measured, independent and observed [*I* > 2σ(*I*)] reflections	87568, 5429, 4773
*R*_int_	0.041
(sin θ/λ)_max_ (Å^−1^)	0.758

Refinement	
*R*[*F* ^2^ > 2σ(*F* ^2^)], *wR*(*F* ^2^), *S*	0.023, 0.057, 1.06
No. of reflections	5429
No. of parameters	199
H-atom treatment	H-atom parameters constrained
Δρ_max_, Δρ_min_ (e Å^−3^)	0.83, −0.46

Computer programs: *APEX3* and *SAINT* (Bruker, 2018 ▸), *SHELXT* (Sheldrick, 2015*a* ▸), *SHELXL2018* (Sheldrick, 2015*b* ▸), *ORTEP-3 for Windows* (Farrugia, 2012 ▸) and *PLATON* (Spek, 2020 ▸).

## supplementary crystallographic information

### Crystal data

**Table:**

C_14_H_8_Br_2_FN_3_O_2_	*D*_x_ = 1.913 Mg m^−^^3^
*M**_r_* = 429.05	Mo *K*α radiation, λ = 0.71073 Å
Orthorhombic, *P**b**c**n*	Cell parameters from 9970 reflections
*a* = 14.8700 (4) Å	θ = 2.5–34.3°
*b* = 15.2915 (4) Å	µ = 5.46 mm^−^^1^
*c* = 13.1030 (4) Å	*T* = 100 K
*V* = 2979.42 (14) Å^3^	Block, light orange
*Z* = 8	0.59 × 0.26 × 0.20 mm
*F*(000) = 1664	

### Data collection

**Table:**

Bruker AXS D8 QUEST Photon III detector diffractometer	5429 independent reflections
Radiation source: fine-focus sealed X-Ray tube	4773 reflections with *I* > 2σ(*I*)
Graphite monochromator	*R*_int_ = 0.041
Detector resolution: 7.31 pixels mm^-1^	θ_max_ = 32.6°, θ_min_ = 2.5°
φ and ω shutterless scans	*h* = −22→22
Absorption correction: multi-scan (SADABS; Krause *et al.*, 2015)	*k* = −23→23
*T*_min_ = 0.047, *T*_max_ = 0.115	*l* = −19→19
87568 measured reflections	

### Refinement

**Table:**

Refinement on *F*^2^	Primary atom site location: structure-invariant direct methods
Least-squares matrix: full	Secondary atom site location: difference Fourier map
*R*[*F*^2^ > 2σ(*F*^2^)] = 0.023	Hydrogen site location: inferred from neighbouring sites
*wR*(*F*^2^) = 0.057	H-atom parameters constrained
*S* = 1.06	*w* = 1/[σ^2^(*F*_o_^2^) + (0.0274*P*)^2^ + 1.6564*P*] where *P* = (*F*_o_^2^ + 2*F*_c_^2^)/3
5429 reflections	(Δ/σ)_max_ = 0.005
199 parameters	Δρ_max_ = 0.83 e Å^−^^3^
0 restraints	Δρ_min_ = −0.46 e Å^−^^3^

### Special details

**Table:**

Geometry. All esds (except the esd in the dihedral angle between two l.s. planes) are estimated using the full covariance matrix. The cell esds are taken into account individually in the estimation of esds in distances, angles and torsion angles; correlations between esds in cell parameters are only used when they are defined by crystal symmetry. An approximate (isotropic) treatment of cell esds is used for estimating esds involving l.s. planes.
Refinement. Refinement of F^2^ against ALL reflections. The weighted R-factor wR and goodness of fit S are based on F^2^, conventional R-factors R are based on F, with F set to zero for negative F^2^. The threshold expression of F^2^ > 2sigma(F^2^) is used only for calculating R-factors(gt) etc. and is not relevant to the choice of reflections for refinement. R-factors based on F^2^ are statistically about twice as large as those based on F, and R- factors based on ALL data will be even larger.

### Fractional atomic coordinates and isotropic or equivalent isotropic displacement parameters (Å^2^)

**Table:**

	*x*	*y*	*z*	*U*_iso_*/*U*_eq_	
Br1	0.62265 (2)	0.95963 (2)	0.16792 (2)	0.02193 (4)	
Br2	0.64744 (2)	1.02154 (2)	0.39424 (2)	0.02154 (4)	
F1	0.61074 (8)	0.42468 (7)	0.03550 (8)	0.0350 (2)	
O1	0.79994 (8)	0.81157 (8)	0.37024 (8)	0.0279 (2)	
O2	0.82768 (8)	0.70323 (8)	0.47212 (9)	0.0279 (2)	
N1	0.78022 (8)	0.76388 (8)	0.44249 (9)	0.0204 (2)	
N2	0.61421 (8)	0.77830 (8)	0.26409 (9)	0.0173 (2)	
N3	0.61871 (8)	0.70235 (8)	0.30197 (9)	0.0180 (2)	
C1	0.62788 (9)	0.92776 (9)	0.30578 (10)	0.0172 (2)	
C2	0.62325 (9)	0.84437 (9)	0.33887 (9)	0.0163 (2)	
C3	0.62473 (9)	0.82188 (9)	0.44946 (10)	0.0160 (2)	
C4	0.69718 (9)	0.78201 (9)	0.49868 (10)	0.0167 (2)	
C5	0.69526 (10)	0.75800 (9)	0.60064 (10)	0.0198 (2)	
H5	0.745761	0.730610	0.631466	0.024*	
C6	0.61793 (10)	0.77479 (10)	0.65687 (10)	0.0216 (3)	
H6	0.615605	0.760257	0.727309	0.026*	
C7	0.54428 (10)	0.81279 (10)	0.60985 (10)	0.0221 (3)	
H7	0.491119	0.823434	0.648092	0.026*	
C8	0.54741 (10)	0.83559 (9)	0.50689 (10)	0.0195 (2)	
H8	0.495992	0.860845	0.475544	0.023*	
C9	0.61222 (9)	0.63404 (9)	0.22921 (10)	0.0173 (2)	
C10	0.62860 (10)	0.55039 (9)	0.26748 (11)	0.0214 (3)	
H10	0.640277	0.542361	0.338142	0.026*	
C11	0.62783 (11)	0.47892 (10)	0.20219 (13)	0.0253 (3)	
H11	0.639299	0.421601	0.226931	0.030*	
C12	0.60996 (11)	0.49353 (11)	0.10055 (12)	0.0245 (3)	
C13	0.59060 (10)	0.57525 (10)	0.06081 (11)	0.0224 (3)	
H13	0.577105	0.582364	−0.009565	0.027*	
C14	0.59139 (10)	0.64622 (9)	0.12606 (10)	0.0194 (2)	
H14	0.577851	0.703011	0.101002	0.023*	

### Atomic displacement parameters (Å^2^)

**Table:**

	*U*^11^	*U*^22^	*U*^33^	*U*^12^	*U*^13^	*U*^23^
Br1	0.02940 (8)	0.02164 (7)	0.01475 (6)	−0.00253 (5)	−0.00260 (5)	0.00271 (5)
Br2	0.02785 (8)	0.01832 (7)	0.01843 (6)	−0.00056 (5)	0.00111 (5)	−0.00383 (5)
F1	0.0542 (7)	0.0220 (5)	0.0287 (5)	0.0035 (5)	−0.0047 (4)	−0.0101 (4)
O1	0.0232 (5)	0.0403 (6)	0.0201 (5)	0.0021 (5)	0.0046 (4)	0.0009 (4)
O2	0.0267 (5)	0.0279 (6)	0.0292 (6)	0.0120 (5)	−0.0039 (4)	−0.0073 (4)
N1	0.0184 (5)	0.0245 (6)	0.0183 (5)	0.0033 (5)	−0.0014 (4)	−0.0066 (4)
N2	0.0197 (5)	0.0170 (5)	0.0152 (5)	0.0005 (4)	0.0008 (4)	−0.0012 (4)
N3	0.0200 (5)	0.0178 (5)	0.0161 (5)	0.0002 (4)	0.0002 (4)	−0.0008 (4)
C1	0.0205 (6)	0.0179 (6)	0.0134 (5)	0.0009 (5)	0.0000 (4)	−0.0017 (4)
C2	0.0170 (5)	0.0182 (6)	0.0136 (5)	0.0015 (5)	0.0003 (4)	−0.0012 (4)
C3	0.0185 (6)	0.0154 (5)	0.0140 (5)	0.0012 (5)	−0.0003 (4)	−0.0006 (4)
C4	0.0181 (6)	0.0160 (5)	0.0161 (5)	0.0014 (5)	−0.0004 (4)	−0.0026 (4)
C5	0.0236 (6)	0.0192 (6)	0.0166 (5)	0.0031 (5)	−0.0031 (5)	−0.0001 (4)
C6	0.0278 (7)	0.0218 (6)	0.0151 (5)	0.0021 (5)	0.0006 (5)	0.0022 (5)
C7	0.0235 (7)	0.0262 (7)	0.0165 (6)	0.0037 (5)	0.0046 (5)	0.0027 (5)
C8	0.0192 (6)	0.0229 (6)	0.0162 (5)	0.0036 (5)	0.0011 (4)	0.0022 (5)
C9	0.0183 (6)	0.0173 (6)	0.0162 (5)	0.0002 (5)	0.0001 (4)	−0.0007 (4)
C10	0.0258 (7)	0.0192 (6)	0.0191 (6)	0.0013 (5)	−0.0022 (5)	0.0009 (5)
C11	0.0321 (8)	0.0180 (6)	0.0259 (7)	0.0029 (6)	−0.0028 (6)	−0.0009 (5)
C12	0.0289 (7)	0.0213 (6)	0.0232 (7)	0.0004 (6)	−0.0013 (5)	−0.0063 (5)
C13	0.0273 (7)	0.0225 (7)	0.0175 (6)	−0.0003 (6)	−0.0012 (5)	−0.0027 (5)
C14	0.0224 (6)	0.0189 (6)	0.0168 (5)	−0.0006 (5)	0.0001 (5)	0.0001 (4)

### Geometric parameters (Å, º)

**Table:**

Br1—C1	1.8725 (13)	C6—C7	1.384 (2)
Br2—C1	1.8667 (13)	C6—H6	0.9500
F1—C12	1.3546 (17)	C7—C8	1.3942 (18)
O1—N1	1.2304 (17)	C7—H7	0.9500
O2—N1	1.2284 (16)	C8—H8	0.9500
N1—C4	1.4641 (18)	C9—C10	1.3953 (19)
N2—N3	1.2647 (16)	C9—C14	1.3992 (19)
N2—C2	1.4138 (17)	C10—C11	1.388 (2)
N3—C9	1.4175 (17)	C10—H10	0.9500
C1—C2	1.3486 (19)	C11—C12	1.376 (2)
C2—C3	1.4895 (18)	C11—H11	0.9500
C3—C8	1.3900 (19)	C12—C13	1.384 (2)
C3—C4	1.3958 (18)	C13—C14	1.382 (2)
C4—C5	1.3859 (18)	C13—H13	0.9500
C5—C6	1.390 (2)	C14—H14	0.9500
C5—H5	0.9500		
			
O2—N1—O1	123.62 (13)	C6—C7—H7	119.7
O2—N1—C4	117.94 (13)	C8—C7—H7	119.7
O1—N1—C4	118.41 (12)	C3—C8—C7	120.92 (13)
N3—N2—C2	112.28 (11)	C3—C8—H8	119.5
N2—N3—C9	114.15 (11)	C7—C8—H8	119.5
C2—C1—Br2	122.32 (10)	C10—C9—C14	120.52 (13)
C2—C1—Br1	123.65 (10)	C10—C9—N3	114.96 (12)
Br2—C1—Br1	113.92 (7)	C14—C9—N3	124.52 (12)
C1—C2—N2	117.24 (12)	C11—C10—C9	119.93 (14)
C1—C2—C3	122.03 (12)	C11—C10—H10	120.0
N2—C2—C3	120.71 (12)	C9—C10—H10	120.0
C8—C3—C4	117.01 (12)	C12—C11—C10	118.05 (14)
C8—C3—C2	118.66 (12)	C12—C11—H11	121.0
C4—C3—C2	124.19 (12)	C10—C11—H11	121.0
C5—C4—C3	123.04 (13)	F1—C12—C11	118.75 (14)
C5—C4—N1	116.88 (12)	F1—C12—C13	117.83 (13)
C3—C4—N1	120.08 (12)	C11—C12—C13	123.42 (14)
C4—C5—C6	118.63 (13)	C14—C13—C12	118.34 (13)
C4—C5—H5	120.7	C14—C13—H13	120.8
C6—C5—H5	120.7	C12—C13—H13	120.8
C7—C6—C5	119.75 (12)	C13—C14—C9	119.68 (13)
C7—C6—H6	120.1	C13—C14—H14	120.2
C5—C6—H6	120.1	C9—C14—H14	120.2
C6—C7—C8	120.61 (13)		
			
C2—N2—N3—C9	−178.50 (11)	C3—C4—C5—C6	−0.4 (2)
Br2—C1—C2—N2	−175.87 (9)	N1—C4—C5—C6	179.45 (13)
Br1—C1—C2—N2	0.19 (18)	C4—C5—C6—C7	1.6 (2)
Br2—C1—C2—C3	5.92 (19)	C5—C6—C7—C8	−1.0 (2)
Br1—C1—C2—C3	−178.02 (10)	C4—C3—C8—C7	2.0 (2)
N3—N2—C2—C1	173.96 (13)	C2—C3—C8—C7	177.74 (13)
N3—N2—C2—C3	−7.80 (18)	C6—C7—C8—C3	−0.9 (2)
C1—C2—C3—C8	75.95 (18)	N2—N3—C9—C10	172.10 (13)
N2—C2—C3—C8	−102.20 (16)	N2—N3—C9—C14	−7.8 (2)
C1—C2—C3—C4	−108.63 (17)	C14—C9—C10—C11	2.6 (2)
N2—C2—C3—C4	73.22 (18)	N3—C9—C10—C11	−177.28 (14)
C8—C3—C4—C5	−1.4 (2)	C9—C10—C11—C12	−0.5 (2)
C2—C3—C4—C5	−176.84 (13)	C10—C11—C12—F1	178.69 (15)
C8—C3—C4—N1	178.78 (12)	C10—C11—C12—C13	−1.7 (3)
C2—C3—C4—N1	3.3 (2)	F1—C12—C13—C14	−178.71 (14)
O2—N1—C4—C5	26.58 (18)	C11—C12—C13—C14	1.6 (2)
O1—N1—C4—C5	−151.37 (13)	C12—C13—C14—C9	0.5 (2)
O2—N1—C4—C3	−153.54 (13)	C10—C9—C14—C13	−2.6 (2)
O1—N1—C4—C3	28.51 (19)	N3—C9—C14—C13	177.24 (13)

### Hydrogen-bond geometry (Å, º)

**Table:**

*D*—H···*A*	*D*—H	H···*A*	*D*···*A*	*D*—H···*A*
C6—H6···O1^i^	0.95	2.51	3.3244 (18)	144

Symmetry code: (i) −*x*+3/2, −*y*+3/2, *z*+1/2.
